# News and Miscellany

**Published:** 1881-04

**Authors:** 


### NEWS AND MISCELLANY.

Mustang Liniment is composed of oleic acid, aqua ammonia, petroleum, and naphtha.

Bi-Carbonate of Potassium in Rheumatism in cattle and dogs is recommended as preferable to salicylic acid by Prof. Robertson, of Edinburgh.

A British Congress of Veterinary Surgeons.—It is proposed to hold a congress of veterinary surgeons in London this year, to discuss matters of interest to the veterinary profession.

Social Recognition of Veterinary Surgeons.—English veterinarians are elated because the Prince of Wales has invited one of their number to dinner, and has thus given social recognition to the profession.

A Veterinary College for Ireland.—A petition has been forwarded to the Lord-Lieutenant asking for a subsidy of $50,000, with which to establish a veterinary college in Ireland.

The New York Aquarium.—We regret to announce that the New York Aquarium has been closed on account of lack of support. The last of the chimpanzees at the institution died there on February 12th, of a throat affection.

A New Zoological Garden in New York City.—A company has been organized in this city with the object of constructing a Zoological Garden here that will eclipse anything in the world.

Prize for an Essay on Paraplegia in the Horse.—A prize of 200 rupees has been offered by the Governor-General of India for the best essay on paraplegia in the horse. The prize is open to general competition. All essays must be handed in by October 1st, 1881.

Veterinary Medicine in England.—A bill to regulate the practice of veterinary medicine in England has been drafted and presented to Parliament. It forbids any one to use the title of veterinary surgeon unless he has received a diploma or license from the proper regulating body.

Salicylic Acid in Foot-and Mouth Disease.—The Duke of Brunswick has successfully treated cases of foot-and-mouth disease among his herd of cattle in Prussian Silesia by salicylic acid. A solution of the acid in hot water is applied externally to the mouth and hoofs, and the drug is given internally in doses of one to two drachms six times a day.

Death of a Veterinary Surgeon from Strychnine.—Ernest Traver, of Rhinebeck, N. Y., in the employ of Mr. Lorillard as a veterinary surgeon, died on March 22, from the effects of an overdose of strychnine, which he had taken for a bad cold an hour previous. He was suffering from nervous prostration at the time, and, it is believed, took more of the fatal drug than he designed.

Foot-and-Mouth Disease in New York City.—The steamship France, of the National Line, brought to this city in January last, besides a number of dogs and horses, two bulls and eight Alderney heifers. Mr. James D. Hopkins, who was detailed by Gen. Patrick to inspect the cattle, found that they were affected with epizootic aptha, or "the foot-and-mouth disease." The animals were promptly quarantined, and no harm has resulted. They subsequently recovered from the disease.

An Analysis of the Haas Hog Cholera Remedy, made by the Agricultural Department, gives the following result:

| | |
|---|---|
| Powdered soap | 10 per cent. |
| Potassium carbonate | 5 " |
| Red ochre | 12 " |
| Precipitated chalk | 50 " |
| Quicklime | 10 " |
| Calcined magnesia | 13 " |
| | 100 |

This shows it to be very much like a humbug.

Strange Cattle Disease.—A disease unknown or undiagnosticated has broken out among the cattle at East Watertown. The stock attacked are blooded animals, and were in the very best condition. Several of them have died. They are taken first by an intense coughing, which is followed by a loss of appetite and a shrinkage of milk. When let out where they can have perfect freedom they appear almost crazy, and will bite themselves, tearing out pieces of flesh, and appear to be in the greatest agony. The attention of the State authorities has been called to the matter.

An Epidemic of Spinal Meningitis Among Horses.—There were said to be, a short time ago, thirty dead horses lying on the bank of the Connecticut river, a mile or two below Hartford, Connecticut. They were victims of spinal meningitis. At Wooley's stables, Main street, Hartford, several cases have occurred, and some are now under treatment; so of some other stables thereabout. Only a few stables, as yet, appear to have it. The disease attacks the horses of private owners as well as those of the livery stables. The main treatment seems to be to give physic balls and a mustard plaster, and blanket the horse. Comparatively few die.

Selling Diseased Meat.—A butcher living on West Thirty-Eighth street was recently convicted of selling diseased meat, and sentenced to three months in the Penitentiary, with a fine of $250. There were found in his establishment 220 heads of diseased meat, 7 baskets of putrid meat, and 81 quarters. The meat sold for five cents a pound, and was used in making sausages. Diseased meat is said to be preferred for New York bologna sausage.

Connecticut Veterinary Law.—There is in Connecticut a law which authorizes a board of commissioners to take possession of any domestic animal which is supposed to be sick with a contagious disease, and dispose of it as they may judge best, keeping it, or compelling the owner to keep it, in quarantine until the disease has been sufficiently developed to allow its character to be determined, or making it the subject of veterinary investigation, or killing it.

The Hog Cholera and Trichinosis Scare.—The British Consul at Philadelphia, several weeks ago, wrote home that hog cholera was very prevalent in this country. This report caused great alarm and agitation among American pork dealers. Investigation has apparently shown that the disease is somewhat less prevalent this year than usual. The canard, however, brought out the fact that an enormous number of hogs die from this disease every year, the number in Illinois alone in 1879 being about 300,000.

Rural Physiology—The editors of the *Country Gentleman* received and published the following: "For two weeks there has been an animated discussion in this village as to whether cattle raise their food (hay, straw, &c.) after swallowing it once, and go through the process of mastication. Has a cow or ox the power to raise a cud from the stomach at will? Please give authority on the above subject.—Bovine, *Jefferson, N. Y.*"

Where is this place in which no one knew certainly whether or not cows ruminated? We suggest for the next "animated discussion" the question: Is the prevailing color of the grass in Jefferson, N. Y., a green; and why?

Extent of Pleuro-Pneumonia in the United States.—It has never extended over any considerable area, the eastern parts of Maryland and Pennsylvania being the extreme western, and northern Virginia the extreme southern limit, while no case has ever been reported in any locality in the State of New York farther north than the county of Putnam—about fifty miles from New York city. The efforts made by the State authorities within two years past for the suppression of the disease have been so far successful that all the State of New York is cleared, excepting Kings and Queens counties (on Long Island). Pennsylvania is reported entirely free from it, and the measures adopted in New Jersey and Maryland have prevented its extending beyond the three or four counties in which it has occurred. Still foci of the disease exist, and it may be carried west at any time.

FOOT-AND-MOUTH DISEASE IN ENGLAND.—Foot-and-mouth disease has spread with great rapidity throughout England, and all fairs and markets have been stopped for a time by Order of Council; probably the order will be renewed at its expiration. Ireland is free from the disease, no cases having been reported since the autumn of 1879; and the Irish Veterinary Department has very properly issued an order prohibiting the importation of cattle, sheep, and swine into Ireland from England. Scotland is also free from the disease, and it is probable that English cattle, sheep, and swine will be prohibited from crossing the border, the necessary powers, both with regard to Ireland and to Scotland, having been conferred by recent legislation.

DOES FOOT-AND-MOUTH DISEASE EXIST IN THIS COUNTRY?—This question has been agitated extensively in the columns of the *Live Stock Journal*. It is asserted by certain English veterinarians that we have the disease, because large numbers of cattle have arrived in England from the United States affected with it. According to Dr. Lyman (*Report on Contagious Diseases*) three cases were landed in Liverpool from Boston last July. Mr. Turner, of Tunbridge, England, states that 380 head of cattle with the disease have been brought from the United States to England. But he does not state the time. On the other hand, it is asserted by the *Live Stock Journal* that there are no known cases of the disease in the country now, or at least in the West; and it is very unlikely that so contagious an affection would exist there without its making some stir. Our correspondent, Dr. Hopkins, is, as will be seen by his letter, decidedly of the opinion that foot-and-mouth disease does prevail in the United States. And the evidence in favor of this view is very strong.

THE BROWN INSTITUTION.—The "Brown" Institution is at Lambeth, Eng., and is practically a hospital for dogs, horses, cattle, and other animals. It is a large establishment, and between two and three thousand "patients" come under treatment every year. Of the 2,465 animals treated there last year, 1,637 were horses, 60 donkeys, 527 dogs, 100 cats, 10 goats, 2 pigs, and 129 smaller animals. Of these, 76 were admitted as in-patients, while the rest were treated entirely as out-patients. A course of five lectures is delivered annually, usually before Christmas. Last year Dr. Greenfield, the professor superintendent, chose for his subject the pathology of pyæmia, septicæmia and anthracoid diseases. This year he gives the results of his further investigations into anthrax and allied diseases in man and the lower animals. His description of woolsorter's disease was most interesting, and is likely to attract considerable attention. The course is illustrated by microscopical demonstrations, photographs, pathological specimens thrown on the screen with the lime light, and so on.—*Therapeutic Gazette.*

Clarke on the Uses of the Canine Teeth.—Mr. W. H. Clarke, in his interesting work on "Horses' Teeth," noticed in our previous issue, shows not only a great deal of painstaking research, but occasionally some touches of humor. We quote his remarks upon the uses of the canine teeth or tushes:—"The Canine Teeth (*laniarii dentes*), comparatively speaking, are of little practical use; at least they are of little use to the modern horse. They have been much reduced in size during the evolution of the horse, and, if Darwin's theory is correct, are probably 'in the course of ultimate extinction.' They distinguish the sex, it is true, but their loss would not be felt on that account. The horse sometimes uses them in tearing bark from trees, for he is by instinct his own (botanical) doctor, and the bark is his medicine. The sharp points of the tushes penetrate the bark more readily than the incisors, and apparently the horse wishes to save his incisors, thus showing his horse-sense. Their nature is different from that of the other teeth, for the incisors and grinders grow till old age. This is not the case with the tushes, and, further, they are never in apposition (superposed), and consequently do not wear one another."

Congress and Veterinary Medicine.—An effort was made at the last session of Congress to create a National Commission for investigating the contagious diseases of animals. The Agricultural Department had one scheme, which, of course, placed the whole matter in its charge. The Western men, on the other hand, wished to have the matter under the charge of a separate commission. It is the opinion of our Western stock breeders, as evidenced by the statements of the *National Live Stock Journal*, that the Commissioner of Agriculture does not know how to investigate contagious diseases among animals. His veterinary assistants, we infer from the same authority, are sunk in professional ignorance, and are chiefly desirous of proving that contagious pleuro-pneumonia exists, or has existed, in Western cattle; also, that foot-and-mouth disease does prevail in the United States. The *National Live Stock Journal* is very much set in the conviction that there are not any, and shall not be any, contagious diseases among Western cattle. Towards this view of the matter, which the journal referred to has so far sustained with great ability and success, the Commissioner of Agriculture and his agents do not feel so kindly. We do not express any opinion here on the merits of the controversy. It is enough to know that it contributed to the unfortunate result of killing the bill before Congress for further investigations of contagious disease. The sole act of Congress favoring veterinary medicine was the printing 50,000 copies of the Commissioner's special report on contagious diseases. It forms a beautiful comment on the intelligence in this direction of our national legislators.

Trichinosis and American Pork.—The discovery of trichinae in American pork imported into France, has so alarmed that government that they have prohibited its importation. This cuts off the exportation of 76,000,000 pounds of pork per year from America. Italy, Spain, Portugal, Germany, Russia, and Greece, have made the same restrictions.

The Swiss government has become alarmed also, and it recently undertook an investigation of the matter. As a result of this, it was concluded that the danger of trichinosis from the source in question was very slight. No authentic instance of trichinosis from American pork imported into Europe is recorded. Switzerland, Belgium, and England, have all refused to follow the example of France.

These countries have, undoubtedly, shown the most judgment and good sense in thus refraining from yielding to a senseless panic.

Nevertheless, American pork dealers should remember that their trade is still on a precarious footing. It has been proved that trichinae do exist in American swine, and that to a large amount. Should trichinosis be actually induced by these in any European country, the injury caused by the action of France would be repeated, and, perhaps, on a more extensive and permanent scale.

Let the pork dealers look to the source of the evil, therefore. Let them find some way to keep trichinae out of pork, or to keep the trichinous pork out of the market.

Columbia Veterinary College—Fourth Annual Commencement.—The Fourth Annual Commencement of the Columbia Veterinary College was held at Chickering Hall, April 7th, 1881. A large audience was present, and showed evidence of much interest in the exercises. The stage was decorated with flowers, and the music was excellent. Professor Louis H. Laudy presided. The Introductory Address was delivered by Dr. T. E. Satterthwaite, and was a very interesting and carefully prepared effort. Prof. E. S. Bates, Dean of the College, conferred the degrees, and the award of prizes was made by Prof. A. S. Heath.

The Valedictory Address, which was exceptionally good, was pronounced by M. L. Frey, D.V.S.

Prizes were awarded as follows:

Anatomical Prize to E. J. Peck, D.V.S.; Pathological Prize to George H. Parkinson, D.V.S.; Silver Medal to E. A. MacLellan; Gold Medal to George H. Parkinson, D.V.S.

The exercises were closed by addresses given by S. D. Sewards, Esq., and Rev. J. H. Rylance.

The following is the list of graduates:

J. H. Dancer, D.V.S., Newark, N. J.; Charles Dunne, D.V.S., New York; Mark L. Frey, D.V.S., New York; George H. Parkinson, D.V.S., Middletown, Conn.; E. Judson Peck, D.V.S., New York; Emile M. C. Savale, D.V.S., Orange, N. J.; Theo. Simons, D.V.S., New York; H. S. Vanderhoff, M.D., D.V.S., Williamsburg, N. Y.; John Campbell Wallace D.V.S., New York.

The following students received the Junior Certificate:

E. S. Breeder, G. F. Bowers, E. F. Dowd, E. W. Douglass, E. M. Fitzgerald, D. Johnston, E. A. MacLellan, P. Newman, W. Soula, F. J. Smith, H. W. Tolles.

The session just closed is said to have been the most satisfactory and successful one since the organization of the College. Already there is felt the need of enlarged accommodations. The interest in Comparative Medicine constantly deepens, and the more intelligent and discerning young men see both honor and pecuniary reward in the practice.

